# Cellular Organelles Reorganization During Zika Virus Infection of Human Cells

**DOI:** 10.3389/fmicb.2020.01558

**Published:** 2020-07-08

**Authors:** Cybele C. García, Cecilia A. Vázquez, Federico Giovannoni, Constanza A. Russo, Sandra M. Cordo, Agustina Alaimo, Elsa B. Damonte

**Affiliations:** ^1^Departamento de Química Biológica, Facultad de Ciencias Exactas y Naturales, Universidad de Buenos Aires, Buenos Aires, Argentina; ^2^Instituto de Química Biológica de la Facultad de Ciencias Exactas y Naturales (IQUIBICEN), Consejo Nacional de Investigaciones Científicas y Técnicas-Universidad de Buenos Aires, Ciudad Universitaria, Buenos Aires, Argentina

**Keywords:** Zika virus, promyelocytic leukemia nuclear bodies, mitochondria, lipid droplets, flavivirus

## Abstract

Zika virus (ZIKV) is an enveloped positive stranded RNA virus belonging to the genus *Flavivirus* in the family *Flaviviridae* that emerged in recent decades causing pandemic outbreaks of human infections occasionally associated with severe neurological disorders in adults and newborns. The intracellular steps of flavivirus multiplication are associated to cellular membranes and their bound organelles leading to an extensive host cell reorganization. Importantly, the association of organelle dysfunction with diseases caused by several human viruses has been widely reported in recent studies. With the aim to increase the knowledge about the impact of ZIKV infection on the host cell functions, the present study was focused on the evaluation of the reorganization of three cell components, promyelocytic leukemia nuclear bodies (PML-NBs), mitochondria, and lipid droplets (LDs). Relevant human cell lines including neural progenitor cells (NPCs), hepatic Huh-7, and retinal pigment epithelial (RPE) cells were infected with the Argentina INEVH116141 ZIKV strain and the organelle alterations were studied by using fluorescent cell imaging analysis. Our results have shown that these three organelles are targeted and structurally modified during ZIKV infection. Considering the nuclear reorganization, the analysis by confocal microscopy of infected cells showed a significantly reduced number of PML-NBs in comparison to uninfected cells. Moreover, a mitochondrial morphodynamic perturbation with an increased fragmentation and the loss of mitochondrial membrane potential was observed in ZIKV infected RPE cells. Regarding lipid structures, a decrease in the number and volume of LDs was observed in ZIKV infected cells. Given the involvement of these organelles in host defense processes, the reported perturbations may be related to enhanced virus replication through protection from innate immunity. The understanding of the cellular remodeling will enable the design of new host-targeted antiviral strategies.

## Introduction

Zika virus (ZIKV) is an enveloped positive stranded RNA virus belonging to the genus *Flavivirus* in the family *Flaviviridae*, which includes other relevant pathogenic arboviruses such as dengue virus (DENV), yellow fever virus (YFV), Japanese encephalitis virus (JEV), and West Nile virus (WNV). ZIKV was initially identified in the Zika forest of Uganda in 1947 from a sentinel rhesus monkey (Dick et al., 1952). Since its discovery, the infrequent human infections reported in Africa and Asia were asymptomatic or generally associated with very mild clinical manifestations (Gubler et al., 2017). This situation dramatically changed in more recent years when ZIKV spread across Asia first to the Pacific Islands and was then introduced into Brazil in 2014. From Brazil, ZIKV was rapidly disseminated throughout the Americas and other regions, with more than 80 countries currently reporting ZIKV autochthonous transmission. Unlike other flaviviruses, the major outbreaks caused by the Asian lineage of ZIKV, particularly in Brazil, were associated with severe neurological complications such as high frequency of newborns with congenital microcephaly (Calvet et al., 2016; Mlakar et al., 2016) and an increase in the number of adults presenting the Guillain-Barré syndrome (Brasil et al., 2016; doRosário et al., 2016). Other major concerns include meningo-encephalitis, myelitis, and ocular abnormalities (Carteaux et al., 2016; Mecharles et al., 2016; Ventura and Ventura, 2018). Most ZIKV human infections are transmitted by the *Aedes aegypti* and *Aedes albopictus* mosquitoes; however, human to human transmission can also occur through sexual contact, vertically from mother to fetus, and by blood transfusion (Musso et al., 2015; Tabata et al., 2016). In fact, the congenital neurological malformations and the sexual transmission have turned ZIKV unique among flaviviruses and highlighted the wide viral tropism that is determinant of the significant pathogenesis.

At present, no specific antiviral agents are available for ZIKV treatment. Increasing evidence has accumulated in recent years about the efficacy of host-targeted therapeutics to obtain a wide spectrum drug active against several related viruses through the interference with cellular factors required to complete an infective virus replication cycle (Acosta and Bartenschlager, 2016; García et al., 2018; Saiz et al., 2018). Additionally a host-directed compound has lower potential to select for resistant variants. For this antiviral strategy, the basic aspects of the virus-cell interaction must be elucidated. After binding and entry by receptor mediated endocytosis, the ZIKV genome is translated in a single polypeptide that is cleaved by viral and host proteases into three structural proteins (the capsid C, the premembrane prM, and the envelope E) that are assembled with RNA in the virion, and seven non-structural (NS) proteins (NS1, NS2A, NS2B, NS3, NS4A, NS4B, and NS5) that are involved in viral replication, pathogenesis, and host antiviral response (Shi and Gao, 2017). All the intracellular steps of flavivirus multiplication are associated to cellular membranes and their bound organelles, leading to an extensive host cell reorganization. As previously reported for DENV and other mosquito-borne flaviviruses, the endoplasmic reticulum (ER) plays a central role in ZIKV infection. In mosquito C6/36 cells and diverse mammalian cells, including Vero and human hepatoma and neuronal progenitor cells, major rearrangements of the ER after ZIKV infection were demonstrated (Barreto-Vieira et al., 2017; Cortese et al., 2017; Offerdahl et al., 2017; Rossignol et al., 2017). Noted morphological changes include membrane invaginations, with development of structures integral to RNA replication, designed replication factories, which are surrounded by drastically reorganized microtubules and intermediate filaments. Concomitantly, virus assembly and budding takes place at ER regions proximal to the replication sites.

In addition to the ER, other cellular organelles are morphologically remodeled and functionally perturbed by flaviviruses. Very active research is available for the role of such organelles, like mitochondria, peroxisomes, lipid droplets (LDs), nuclear compartments and others, in DENV infection (Samsa et al., 2009; Carvalho et al., 2012; Jordan and Randall, 2017), but very few studies have been performed with ZIKV. The targeting of cellular organelles in ZIKV infection has been proved by studies of intracellular localization of viral proteins through immunocytochemistry and proteomics analysis (Hou et al., 2017; Coyaud et al., 2018). But the current knowledge of alterations in several organelles parameters, like morphology, content, dynamics and, consequently, their function, as result of ZIKV infection is still scarce. Since the involvement of most organelles in innate immunity and host defense is well known, the characterization of the virus-induced intracellular reorganization is a key step in order to understand and counteract the mechanisms of virus infection.

Promyelocytic leukemia nuclear bodies (PML-NBs) are nuclear membraneless organelles which contain several cellular proteins, among them mainly PML protein, involved in intrinsic antiviral responses against a number of viruses (Borden, 2002; Geoffroy and Chelbi-Alix, 2011; Scherer and Stamminger, 2016; Guion and Sapp, 2020). Our previous studies have shown that PML exerts antiviral activity against the four DENV serotypes. Furthermore, microscopic analysis revealed that PML-NBs are disrupted after DENV infection due to the interaction of NS5 protein and PML protein, contributing to the DENV induced suppression of the host antiviral response (Giovannoni et al., 2015, 2019). Considering that the nuclear localization of 3 viral proteins, C, NS1, and NS5, has been described in ZIKV infected Vero cells through immunocytochemistry observations (Hou et al., 2017), here we extend our studies to explore the impact of ZIKV infection on PML-NB structure. Moreover, we also evaluated the effect on the organization of two cytoplasmic organelles also participating in the host defense, mitochondria and LDs, in ZIKV infected human cells.

## Materials and Methods

### Cells and Virus

Human hepatoma Huh-7 and monkey Vero (ATCC, CCL81) cells were grown in Dulbecco’s Modified Eagle’s medium (DMEM, GIBCO) supplemented with 10 % fetal bovine serum (FBS), 100 IU/ml of penicillin and 100 μg/ml of streptomycin.

Neural progenitor cells (NPCs; ≥90% SOX1+/Nestin+) derived from human pluripotent stem cells (PSCs) under serum-free conditions (Stem Cell Technologies, Catalog # 70901, https://cdn.stemcell.com/media/files/pis/DX21378-PIS_1_1_0.pdf) were grown using neural progenitor medium 2 (Stem Cell Technologies).

Two human retinal pigment epithelial (RPE) cell lines were employed: ARPE-19 (ATCC^®^ CRL-2302^TM^) cell line was kindly provided by Dr. J.G. Galletti and Dr. M. Guzmán (Instituto de Medicina Experimental IMEX, Buenos Aires, Argentina). The human-Telomerase Reverse Transcriptase immortalized RPE cell line (hTERTRPE-1; ATCC^®^CRL-4000^TM^) cell line was gently provided by Dr. C.A. Bueno (IQUIBICEN, Buenos Aires, Argentina). ARPE-19 is a spontaneously arising RPE cell line of male origin that maintains normal karyology as well as structural and functional properties of RPE cells *in vivo* (Dunn et al., 1996). hTERTRPE-1 is a near-diploid human cell line of female origin with a modal chromosome number of 46 in 90% of the cells counted (Rambhatla et al., 2002). Cells were cultured in DMEM supplemented with 10% heat- inactivated FBS, 2.0 mM glutamine, 100 units/ml penicillin, 100 μg/ml streptomycin, and 0.25 μg/ml amphotericin.

The C6/36 mosquito cell line (from *Aedes albopictus*, ATCC CRL-1660), adapted to grow at 33°C, was cultured in L-15 medium (Leibovitz; GIBCO) supplemented with 0.3% tryptose phosphate broth, 0.02% glutamine, 1% MEM non-essential amino acids solution and 10% FBS. All cell lines were authenticated and tested for contamination.

The ARG INEVH116141 strain of ZIKV (ZIKV-AR) was provided by the Instituto Nacional de Enfermedades Virales Humanas “Dr. Julio I. Maiztegui,” Pergamino, Argentina. Virus stocks were prepared in C6/36 cells and titrated by a standard plaque assay in Vero cells.

All work with infectious agents was performed in biosafety level 2 facilities and approved by the Office of Environmental Health and Safety at the School of Sciences, University of Buenos Aires.

### Confocal Immunofluorescence, Imaging and Quantification of PML-NBs and Mitochondria in ZIKV Infected Cells

Immunofluorescence was performed as previously described (Alaimo et al., 2019; Giovannoni et al., 2019). Briefly, NPCs, ARPE-19, and hTERT RPE-1 cells grown on coverslips were infected with ZIKV at a multiplicity of infection (MOI) of 0.1. After 48 h of infection, cells were fixed with paraformaldehyde (PFA) 4%, permeabilized with Triton X-100 0.1% and stained for immunofluorescence. Primary antibodies used were: anti-PML (Santa Cruz Biotechnology, sc-966, 1:300), anti-NS5 (Genetex, GTX133312, 1:300), anti-TOM20 (sc-11415, Santa Cruz Biotechnology, 1:300), and anti-flavivirus E (Abcam, ab155882, 1:300). Secondary antibodies were: Alexa Fluor 488-anti–rabbit/mouse IgG (Thermo Fisher Scientific, Waltham, MA, United States, 1:400) and Alexa Fluor 555-anti–mouse IgG (Thermo Fisher Scientific, 1:400). Finally, coverslips were mounted in Prolong Gold mounting medium with 4’, 6-diamidino-2-phenylindole (DAPI; Thermo Fisher Scientific). Samples were examined under epifluorescence and confocal microscope Olympus IX71 and FV300, respectively, (Olympus Optical Co., Tokyo, Japan) employing an Olympus 60× oil-immersion Plan Apo objective. Digital images were optimized for contrast and brightness using Adobe Photoshop 7.0 Software.

Quantification of the average number of PML-NBs per cell nucleus was performed using the Fiji distribution of ImageJ. Each cell to be counted was selected and the Find Maxima tool was used. 3D reconstruction of PML-NBs in ZIKV-infected NPCs were generated using the Volume Viewer plugin in Fiji. 2.5D intensity plots were generated using Zen Blue software (Carl Zeiss).

To quantify the mitochondrial morphologies, 100 cells/sample were scored and classified as cells exhibiting tubular (normal) and fragmented (small and spherical) mitochondria according to (Alaimo et al., 2019; 2020). Confocal images were subjected to 3D reconstructions through Fiji imaging software by applying “3D Volume” plugin [National Institutes of Health (NIH) Bethesda, MD], according to (Chatel-Chaix et al., 2016).

### Mitochondrial Membrane Potential Analysis

ARPE-19 cells grown on coverslips were infected with ZIKV (MOI of 0.1). At 48 h post infection (p.i.), supernatant was discarded, cells were washed twice with PBS and incubated with the cell-permeant mitochondria-specific fluorescent reagent MitoTracker Red CMXRos (150 nM in serum-free media, 30 min, 37°C). Accordingly to manufacturer’s indications, this probe stains mitochondria in live cells and its accumulation is dependent upon membrane potential. Afterwards, cells were washed twice with PBS and fixed with 4% PFA (20 min at room temperature). Finally, cells were washed with PBS and mounted on glass slides. Samples were examined under a fluorescence microscope Olympus IX71 equipped with objective lens 60X/1.43 oil (λex: 543/20 nm; λem: 593/40 nm). Capture selected images were optimized for contrast and brightness using Adobe Photoshop 7.0 Software.

### Lipid Droplet Count and Volume Determinations

Huh-7 cells grown on coverslips were infected with ZIKV at a MOI of 0.1. At 24 h p.i., cells were fixed with PFA 4%, permeabilized with Triton X-100 0.1% and stained for immunofluorescence. Antibodies used were anti-flavivirus E (Abcam, ab155882, 1:300) and Alexa Fluor 488-anti–rabbit IgG (Thermo Fisher Scientific, 1:400). The LDs specific probe used was HCS LipidTOX^TM^ Deep Red Neutral Lipid Stain (H34477, Thermo Fisher, 1:250).

*Z*-stacks were acquired in a confocal Olympus – FV1000, at 500 nm intervals and analyzed using Fiji software. First, ZIKV infected cells were selected using green channel. This channel was used to select the regions of interest (ROIs) pertaining to individual cells in images of both non-infected and infected cell cultures (Supplementary Figures 1A,I). In the case of non-infected cultures, cell autofluorescence signal was enough to detect individual cells (Supplementary Figure 1A, see inset). This channel was filtered with a “Gaussian blur” filter with a radius of 40 and binarized using a “Mean” threshold, which sets the threshold as the mean gray level of the stack (Supplementary Figures 1B,C,J,K). When needed, the resulting binary mask was refined with “Close,” “Fill Holes” or “Watershed” algorithms in order to ensure that the whole cell surface was being selected (Supplementary Figures 1D,L). The final binary mask was analyzed with the “Find Particles” plugin to obtain the ROIs corresponding to each cell in every plane of the *Z*-stack (Supplementary Figures 1E,M). Unspecific background was subtracted from LipidTox channel (Supplementary Figures 1F,N) and “3D Object Counter” plugin was used to select LDs (Supplementary Figures 1G,O). Each LDs was assigned to a cell using “Intensity Measurements 2D/3D in MorphoLibJ” library. Volumes of all LDs in the same cell were added to determine total LDs volume. In order to determine the number of LDs per cell, “Find Maxima” plugin was used, with a prominence <45 (Supplementary Figures 1H,P).

### Statistical Analysis

Experiments were carried out in triplicate unless otherwise stated. Experimental comparisons between treatments were made by *t*-Student’s test with statistical significance set at *p* < 0.05. All analyses were carried out with GraphPad Prism 5 software (San Diego, CA, United States).

## Results

### ZIKV Infection Promotes PML-NBs Disruption

Promyelocytic leukemia nuclear bodies are highly dynamic nuclear structures that involve the entry of enzymes and substrates to carry out various cellular functions. Without PML-NBs, these processes would be less efficient or not possible at all in the cytoplasm. Importantly, PML-NBs have shown to limit viral replication in several viral models through multiple mechanisms, and in consequence, many viruses encode products that modify the localization or eliminate PML-NBs in cultured cells (Geoffroy and Chelbi-Alix, 2011; Giovannoni et al., 2015; Brown et al., 2016; Schilling et al., 2017). PML is the major structural component of PML-NBs, and their stability depends on PML presence. In this context, any PML role in ZIKV infection is still unknown.

**FIGURE 1:**
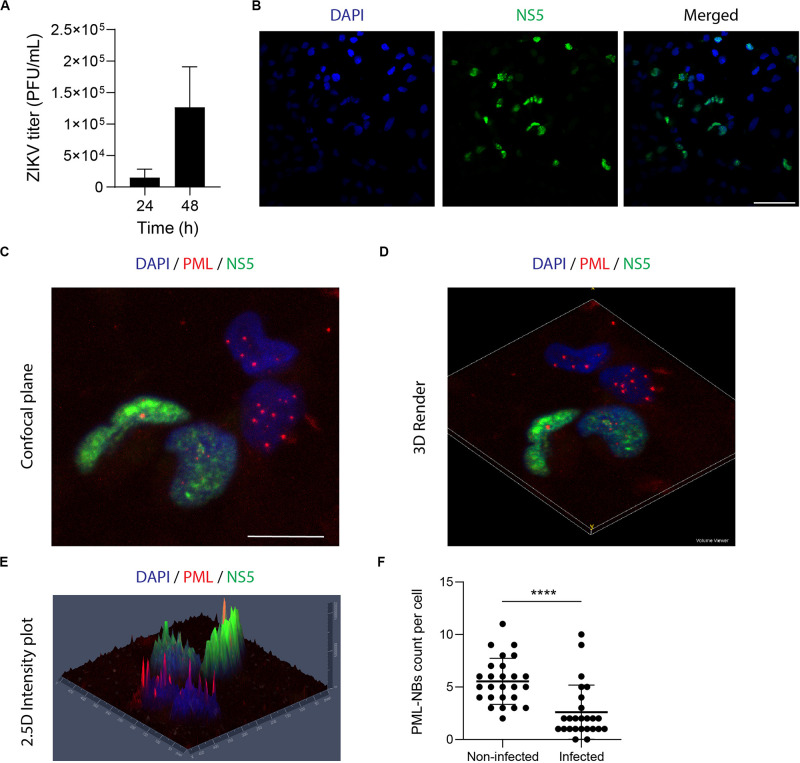
ZIKV infection reduces the number of PML-NBs in NPCs. **(A)** NPCs were infected with ZIKV and supernatants were harvested for plaque assay at 24 h and 48 h p.i. Data represent the mean ± SD (*n* = 3 independent experiments). **(B)** NPCs were infected with ZIKV for 48 h, fixed, and stained against NS5 (green). Nuclei were counterstained with DAPI. Scale bar: 50 μm. **(C)** NPCs were infected with ZIKV for 48 h, fixed and stained against NS5 (green), and PML (red). Nuclei were counterstained with DAPI. Scale bar: 10 μm. **(D)** 3D reconstruction of PML-NBs in ZIKV-infected NPCs was generated using the Volume Viewer Plugin in Fiji. **(E)** 2.5D intensity plot of PML-NBs in ZIKV-infected NPCs was generated using Zen Blue Software. Individual peaks represent absolute signal intensities of each pixel. **(F)** Quantification of the average number of PML-NBs in ZIKV-infected and non-infected NPCs. Data represent the mean ± SD (*n* = 25 cells per condition). ****p < 0.001 *p* value was determined by a two-sided Student’s *t*-test.

It is well established that ZIKV replicates in NPCs (Qian et al., 2016) provoking alterations of cellular pathways which are thought to promote Zika’s congenital syndrome brain abnormalities (Calvet et al., 2016; Mlakar et al., 2016; Yockey et al., 2016; Chavali et al., 2017). Since those studies were performed with different ZIKV strains, we sought to determine the NPCs cultures permissiveness to our viral model. Thus, NPCs were infected with ZIKV-AR and supernatants were collected at 24 and 48 h p.i., for extracellular viral particle quantification. Also, viral antigen detection was performed by IFI. At 24 h p.i. viral yield resulted in 1 × 10^4^ PFU/ml, but no significant viral protein expression was quantified. However, at 48 h p.i. viral titer increased to1.5 × 10^5^ PFU/ml and accordingly, 47% of NPCs were expressing the NS5viral protein (Figures 1A,B). In order to explore the PML-NBs distribution in ZIKV infected NPCs, double immunofluorescence studies were performed employing antibodies against the NS5 viral protein, of nuclear localization, and against PML. Figure 1C shows representative images from 48 h infected NPCs cultures. 3D reconstruction of high-resolution confocal Z-series images (Figure 1D) showing PML (red channel) and NS5 (green channel) and a meticulous analysis using Zen Blue Software (Figure 1E) allowed to quantify the number of PML-NBs in the ZIKV infected cell samples (Figure 1F). As can be seen in NS5 negative ZIKV cells, the typical punctuated nuclear staining pattern of PML-NBs corresponding to the red channel, with an average number of 5 PML-NBs/cell in these control cells, was found. In contrast, a clear and significant decrease in the number of these structures was found in ZIKV infected cells averaging 2.5 PML-NBs/cell. These data are summarized in Figure 1F accounting for a 52 % reduction of PML-NBs in ZIKV infected NPCs cultures in comparison to non-infected ones.

### ZIKV Infection Disturbs Mitochondrial Dynamics

Mitochondria are highly dynamic organelles that can fuse and divide during cell life cycle, and these processes are regulated by a tight equilibrium between two antagonistic events: fusion and fission. This balance plays a critical role in preserving functional mitochondria and consequently, in cell physiology (Pernas and Scorrano, 2016; Giacomello et al., 2020), and can get easily disturbed under intracellular or extracellular stresses. The exploration of the interplay between those stressors, the mitochondrial dynamics and the mechanisms that coordinates how cells respond to them is essential for the understanding of the turnover from health to disease (Eisner et al., 2018). Not surprisingly, it has been suggested that viral infections employ mitochondrial dynamics alteration for the maintenance of persistent infection (Khan et al., 2015; Kim et al., 2018).

Ocular abnormalities present in microcephalic infants with presumed ZIKV congenital disease include conjunctivitis, changes in retinal pigmentation, chorioretinal atrophy, optic nerve abnormalities, hemorrhagic retinopathy and abnormal retinal vasculature (Roach and Alcendor, 2017). In addition, ZIKV preferentially infects Müller and RPE cells, impairing their neurotrophic functions, and eliciting retinal inflammatory responses (Zhao et al., 2017). Notably, the RPE localized in the macular area lies in a high oxidative environment, because of its high metabolic demand, reactive oxygen species (ROS) levels, blood flow and mitochondria content (Moine et al., 2018; Dieguez et al., 2019; Alaimo et al., 2020). The permissiveness of the RPE to viral infections makes it a pertinent tissue to explore the host-cell interactions (Simonin et al., 2019).

**FIGURE 2:**
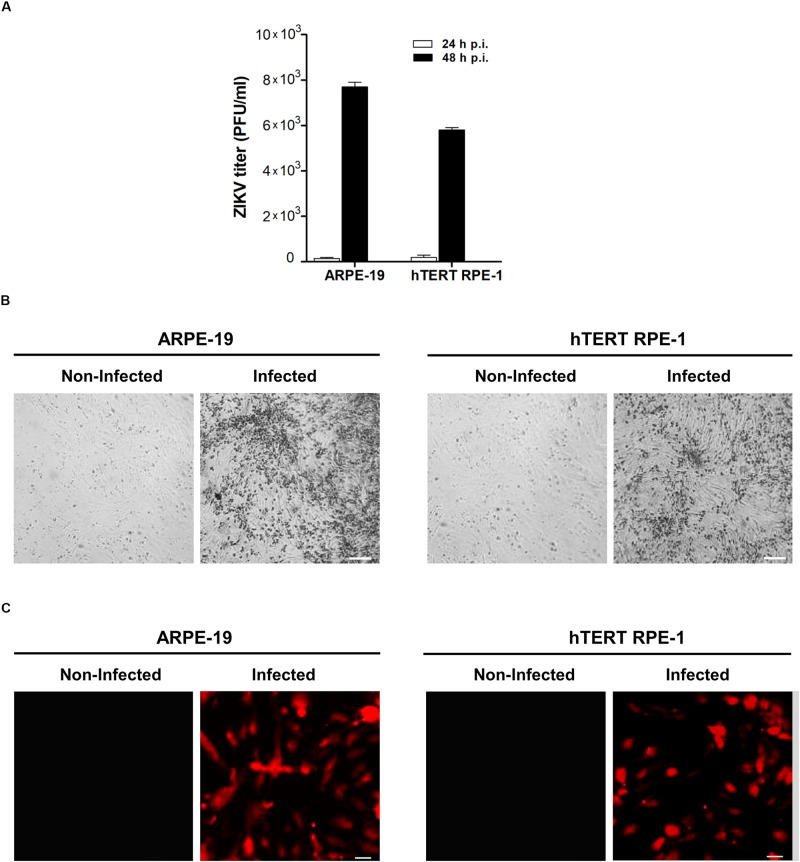
ZIKV infection in human RPE cells. **(A)** ARPE-19 and hTERT RPE-1 cells were infected with ZIKV and infectious virus particles were determined in supernatants at 24 or 48 h p.i., by plaque assay in Vero cells. Data represent the mean ± SD from three independent experiments. **(B)** Morphological signs of cytopathic effects in ZIKV-infected RPE cells at 48 p.i. detected by phase-contrast microscopy. Scale bar: 50 μm. **(C)** ARPE-19 and hTERT RPE-1 cells infected with ZIKV were fixed and stained for flavivirus E protein at 48 h p.i.. Scale bar: 10 μm.

To gain further insight on this interplay, human ARPE-19, and hTERTRPE-1 cell lines were employed to determine the effect of ZIKV on mitochondrial dynamics. Initially, we evaluated the level of viral infection that could be achieved on these cell systems. RPE cell cultures were infected and supernatants were collected at 24 and 48 h p.i. for viral titers quantification by plaque assay. No extracellular viral particles were detected at 24 h p.i. However, 7.8 × 10^3^ PFU/ml, and 6 × 10^3^ PFU/ml were determined at 48 h p.i. on human ARPE-19 and hTERT RPE-1 infected cell lines, respectively, (Figure 2A). In agreement with these results, cytopathic effects were observed under light microscopy only at 48 h p.i. (Figure 2B). At this time, 50% of cells expressing viral antigen were determined by immunofluorescence in both RPE cell cultures (Figure 2C). Taking together these findings, we decided to perform mitochondrial dynamics studies at this time point of infection.

**FIGURE 3:**
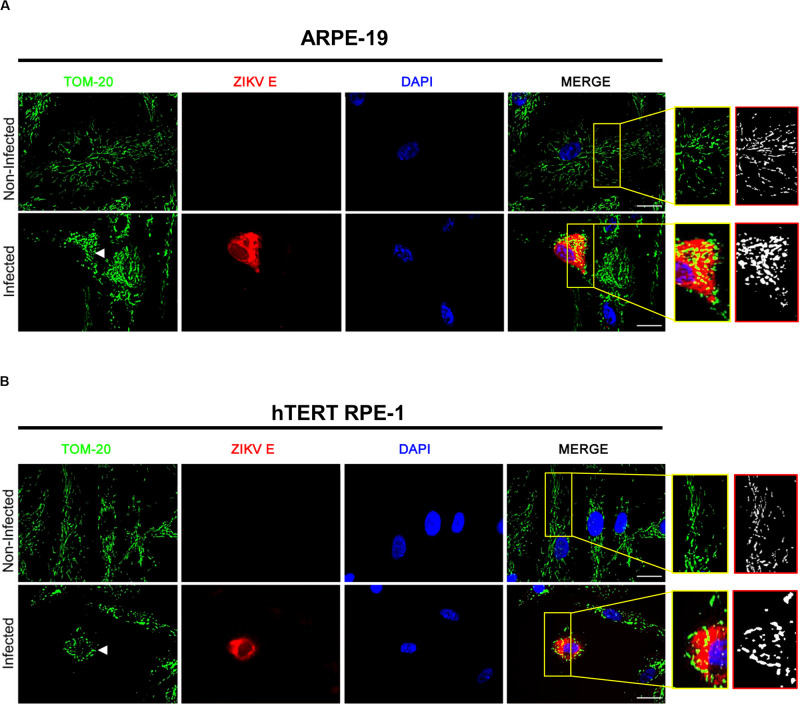
Effect of ZIKV infection on mitochondrial dynamics in RPE cells. ARPE-19 **(A)** and hTERT RPE-1 **(B)** cells were infected with ZIKV at MOI of 0.1 or left uninfected. At 48 h p.i., cells were fixed, permeabilized and mitochondria, viral E glycoprotein and nuclei were stained with anti-TOM-20, 4G2 anti-E mAb, and DAPI, respectively. White arrowhead indicates the branching mitochondrial networks disruption denoted by punctate mitochondria. Insets: for a better visualization of mitochondrial morphology, zoomed areas of grayscale images were pooled to obtain binary images.

ARPE-19 (Figure 3A) and hTERT RPE-1 (Figure 3B) cells were infected with ZIKV and fixed at 48 h p.i.. Immunocytochemical studies of TOM-20 (a central component of TOM, translocase of the outer membrane receptor complex) were performed to analyze mitochondrial morphology. Non-infected ARPE-19 and hTERT RPE-1 cells displayed tubular, filamentous- like mitochondria. On the other hand, ZIKV infection induced a dramatical increase in the population of both RPE cell lines with punctiform fragmented mitochondria (Figures 3A,B).

**FIGURE 4:**
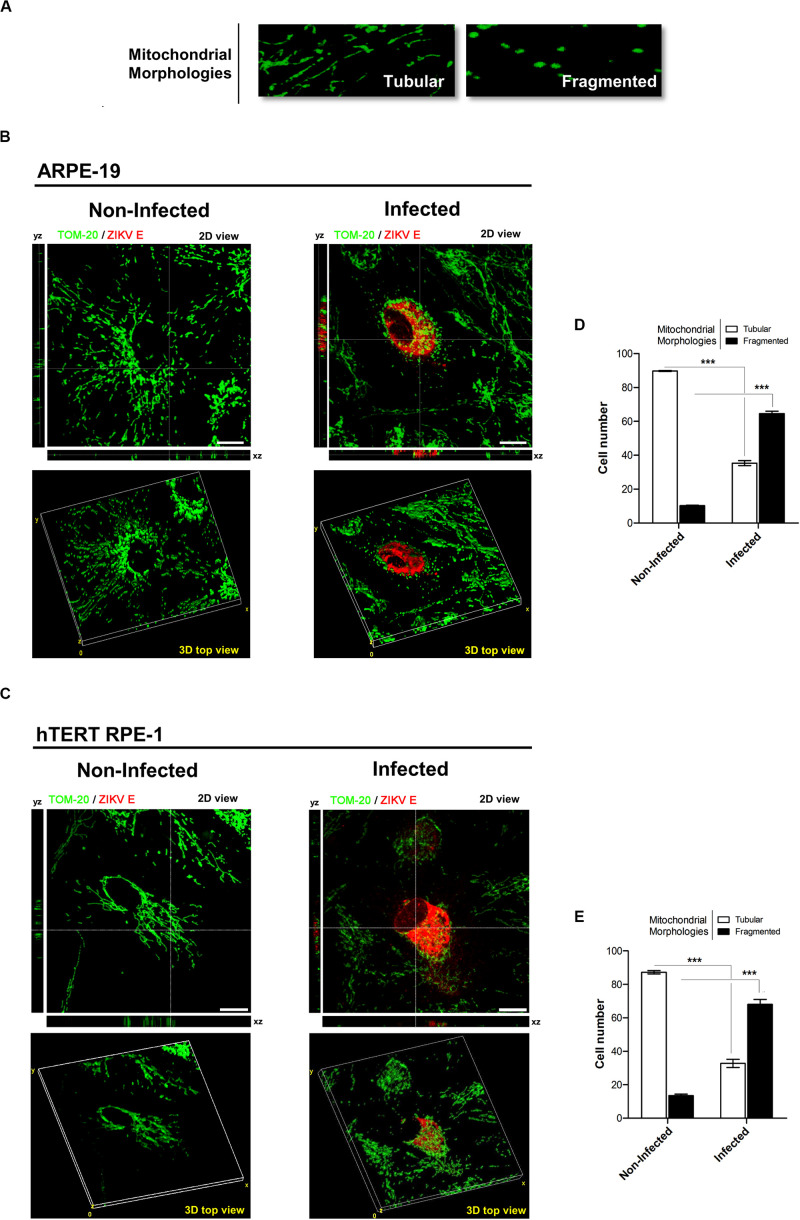
ZIKV infection induces mitochondria fragmentation. **(A)** Mitochondrial morphologies detected: tubular (normal) and fragmented (small and spherical). **(B,C)** Representative confocal *z*-stacks images from non-infected and infected cells used for 3D reconstruction. The crosshairs indicate the positions of the *xz* and *yz* planes. **(B)** ARPE-19, **(C)** hTERT RPE-1. Scale bar: 10 μm. **(D,E)** Quantification of tubular or fragmented mitochondria in infected and non-infected cells; **(D)** ARPE-19, **(E)** hTERTRPE-1. *N* = 100 cells/condition, in quadruplicate. ***p < 0.001 vs. non-infected cells.

In a complementary way, we generated a 3D image reconstruction and volumetric rendering corresponding to samples visualized with fluorescence microscopy. Tubular structures that move in and out of the focal plane can be easily mistaken for individual rod or spherical organelles in conventional imaging (Olichon et al., 2003; Alaimo et al., 2014). Consequently, the stacks acquisition of mitochondrial images along the *Z*-axis of the entire cell provided us a more exhaustive visualization and quality of the morphological alterations that occur in mitochondria of infected cells. In addition, *Z*-stacks acquisition allowed us to establish a more exact morphological classification of these organelles (Figures 4A–C). By this way, an increased cellular population with fragmented mitochondria were quantified in both ARPE-19 (54.4%, *p* < 0.001), and hTERT RPE-1 (54.5%, *p* < 0.001) cells (Figures 4D,E).

**FIGURE 5:**
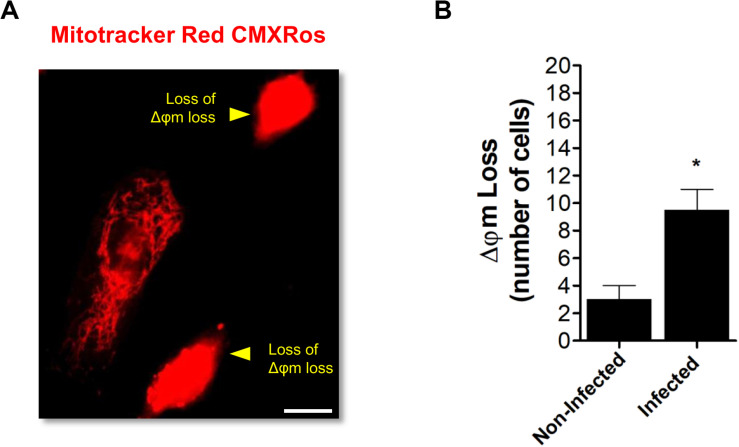
ZIKV-induced mitochondrial dysfunction. **(A)** ARPE-19 cells were infected with ZIKV. At 48 h p.i. cells were stained with MitoTracker Red CMXRos and analyzed by fluorescence microscopy. Cells with loss of mitochondrial transmembrane potential (Δφm, yellow arrowheads) were identified. **(B)** Cells with Δφm loss were scored among 100 cells per condition. Scale bar: 10 μm. Data represent the mean ± SD. *p < 0.05 vs. non-infected.

Finally, a mitochondrial morphodynamic perturbation with a loss of mitochondrial membrane potential (Δφm) was observed in ARPE-19 cells infected with ZIKV (Figures 5A,B). Overall, these analyses demonstrate that ZIKV infection induces an imbalance in fusion/fission equilibrium in favor to the latter event.

### ZIKV Infection Reduces Lipid Droplet Number

Lipid droplets are dynamic intracellular organelles which are required for storing lipids in a cell. They play a major role in energy homeostasis and membrane trafficking (Herker and Ott, 2012). It is very well known that many RNA viruses exploit the LDs energy storing capacity to facilitate their replication (Herker and Ott, 2012). Previous investigations and our data from microarray analysis, which showed the interplay between DENV and lipid pathways, led us to study the LDs pattern changes along the replication of ZIKV (Heaton and Randall, 2011; Martín-Acebes et al., 2016; Vázquez et al., 2019).

**FIGURE 6:**
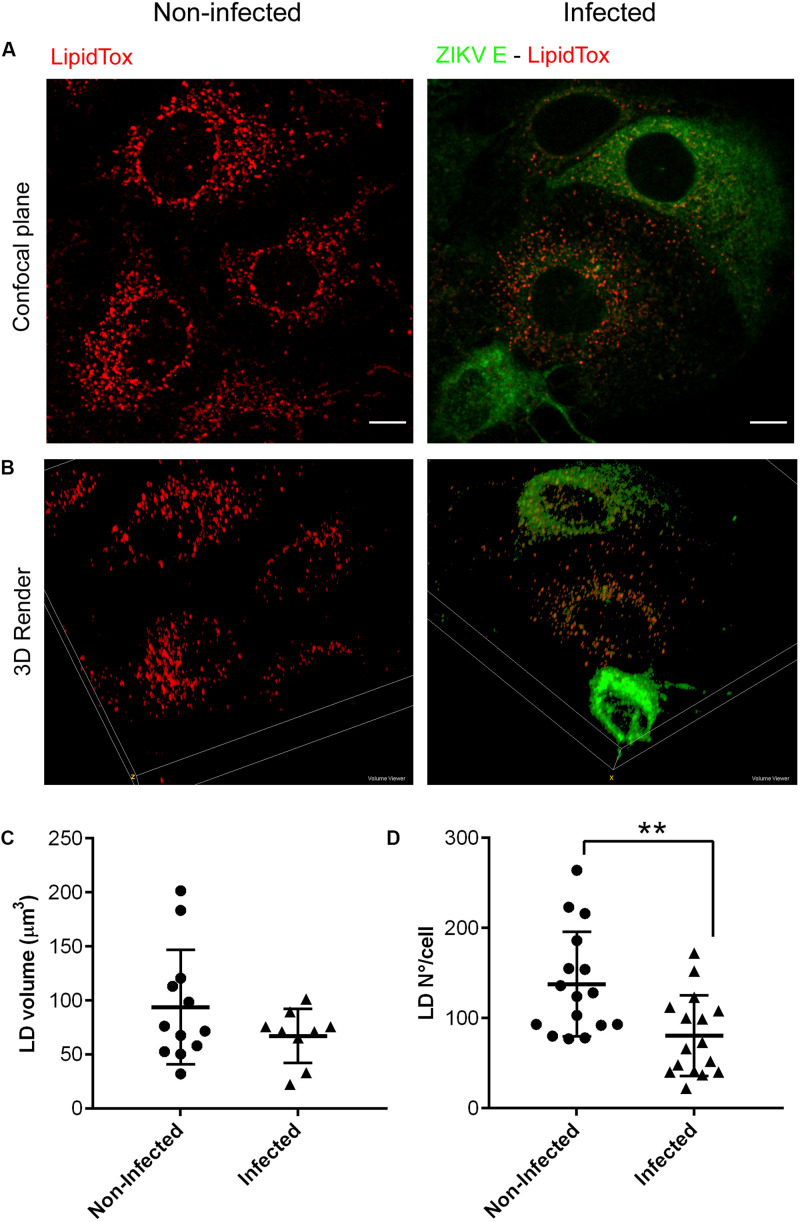
ZIKV infection reduces LDs content. Huh-7 cultures were mock-infected or infected for 24 h. After fixation, LDs were stained with LipidTox (red) and ZIKV infected cells were immunolabeled with anti-E protein (green). Confocal microphotography (upper panel) and 3D render (lower panel) of a non-infected culture **(A)** or a ZIKV infected culture **(B)**. Scale bar: 10 μm. Quantification of LDs number per cell **(C)** and total LDs volume per cell **(D)** in ZIKV-infected and non-infected Huh-7 cultures. Data represent the mean ± SD (*n* = 16 cells per condition for LDs number, and *n* = 10 cells per condition for LDs volume). **p-value ≤ 0.01 (two-sided Student’s *t*-test).

In addition to cytosolic LDs that are present in most other cell types, hepatocytes contain at least two more types of LDs in the lumen of the ER where ZIKV replication occurs, representing the most suitable cellular model to study LDs parameters along flavivirus infection. Different reports have shown opposite results on the LDs modulation exerted by flavivirus infection. Both increased (Samsa et al., 2009) and decreased (Heaton and Randall, 2010,2011) numbers of LDs have been documented using relevant hepatoma cell lines like (HepG2, Huh-7) and also BHK.21 cells. Interestingly, these studies were performed on monolayers infected at high MOI (i.e., 2 or 10) and times ranging from 24 to 72 h p.i. In our study, Huh-7 cell cultures, which are highly permissive to ZIKV infection, were used to evaluate the interplay between LDs and ZIKV infection. Importantly, in order to ensure that the measured effect occurred upon one cycle of viral replication, we infected samples at MOI = 0.1 and analyzed them at 24 h p.i. Therefore, in our assays we consistently worked with an average of 40–50% of positive cells to study the effect on LDs number and content. Double immunofluorescence *Z*-stacks of non-infected (Figure 6A) and infected samples (Figure 6B) showing both ZIKV E protein (green channel) and LDs labeled with LipidTox (red channel) were captured with a confocal microscope. LDs quantification was performed from collected images in both positive and negative ZIKV cells. As can be seen in the histogram shown in Supplementary Figure 1A’s inset, photographs of the green channel in non-infected cultures carry information of cell’s autoflourescence that was enough to detect individual cells. When compared to the histogram shown in Supplementary Figure 1I, it should be noted that scales differ and that the gray values detected in infected cultures are higher than the ones acquired for non-infected cultures, confirming the specificity of the signal. From a detailed inspection under the microscope a decreased number of LDs was seen on those ZIKV infected cells. Extensive image processing and analysis proved a significant decline in LDs number in ZIKV infected cells (Figure 6B, upper panel) when compared with control non-infected cells (Figure 6A, upper panel). A 3D render view of representative fields of both non-infected and infected samples is shown in Figure 6, bottom panels. The differences in LDs enumeration (Figure 6C) appeared to have a correlate with a trend toward a decrease in total LDs volume in infected cells (Figure 6D), suggesting an overall consumption or exhaustion of these organelles.

## Discussion

Microscopes have been the primary scientific instrument in biological sciences and their performance and versatility have improved dramatically over the last 20 years. The ground of microscopy has been particularly fruitful in cell biology studies, including organelle characterization. In this paper we made use of confocal microscopy to uncover new details in the subcellular active reorganization of ZIKV infected cells. For instance, applying 3D reconstruction to focal stacks that can be visualized using volume rendering, we could perform volumetric studies in cellular substructures. The results here reported have shown active morphological alterations and remodeling in nuclear and cytoplasmic organelles, like PML-NBs, mitochondria and LDs of human cells infected with ZIKV.

The involvement of the cell nucleus in the infective process of several RNA viruses has been well demonstrated. Among flaviviruses, the localization of DENV and ZIKV proteins, particularly C, and NS5 proteins, in the nucleus/nucleolus was recognized as indicative of a nucleocytoplasmic trafficking central for virus infection (Hou et al., 2017; Tiwari and Cecilia, 2017). It was recently demonstrated that compounds targeted to the nucleolus structure were inhibitors of ZIKV infection, suggesting a critical nuclear function for viral propagation (Tokunaga et al., 2020). The PML-NBs are other subnuclear components which can act as a target for viruses to escape the antiviral signaling response (Geoffroy and Chelbi-Alix, 2011; Scherer and Stamminger, 2016). In previous studies, the role of PML-NBs in DENV infection was reported demonstrating an interaction between the PML isoforms with the viral protein NS5 that lead to PML-NB degradation in the infected cell (Giovannoni et al., 2015, 2019). Here we analyzed the alterations in the PML-NB structure after infection of NPCs with ZIKV. The punctate staining of NS5 was found in the nucleus of infected cells whereas a significant reduction in the number of PML-NBs was determined. PML-NB structures could be interfered through subcellular translocation into the cytoplasm or disturbance of the nuclear structure with dispersion patterns or/and declining expression. Our results show for the first time that ZIKV infection promotes the breakdown and exhaustion of PML-NBs, corroborating the apparent participation of these subnuclear organelles in the flavivirus life cycle.

Mitochondria are organelles found in the cytoplasm that act as a common platform for the execution of a variety of cellular functions in normal or infected cells. In this sense, mitochondria play central roles as a hub of innate immune signaling and energetic metabolism establishing the major causes of viral pathogenesis (Anand and Tikoo, 2013; Pourcelot and Arnoult, 2014; Kim et al., 2018). The viral strategy to avoid the mechanism of antiviral signaling associated with mitochondria is one of the paradigms of virus-mitochondrial interactions. Until now, studies describing the relationship between flavivirus infection and alterations in mitochondrial dynamics are mainly focused on DENV. Notably, while the existing reports all conclude the occurrence of mitochondrial fusion and fission imbalance in infected cells, there are controversies over which process is favored. Yu et al. (2015) demonstrated that the four DENV serotypes blocked mitochondrial fusion to manipulate the outcome of infection in human lung carcinoma A549 cells. In contrast, other authors reported that DENV serotypes 1, 3, and 4 promote mitochondria fusion in the hepatocarcinoma cell line Huh-7, suggesting that the generation of elongated mitochondria would favor viral replication and dampen activation of the interferon response (Chatel-Chaix et al., 2016; Barbier et al., 2017). Notably, as far as we know, only Chatel-Chaix et al. (2016) mentioned that two ZIKV strains, belonging to the Asian and the African lineage, change the mitochondrial dynamics statement by inducing organelle fusion in Huh-7 cells. In the present work, we analyzed the infection of two lines of human retinal cells, according to their relevance due to the ocular abnormalities associated to ZIKV pathology in humans, with a ZIKV strain of the Asian lineage. In these conditions, we demonstrated that ZIKV shifts the balance of mitochondrial dynamics toward fission in both infected male and female derived-RPE cells in a similar way. At present, all these contrasting results cannot be explained, but it cannot be discarded that both host cell type and the virus source may affect the outcome of the virus-induced alterations in an organelle participating in innate immunity and cell death cascades.

Regarding to cellular structures linked to lipid metabolism, the involvement of LDs in flavivirus infection has been already described for DENV. Some groups have reported an increase in LDs during DENV infection (McLauchlan, 2009; Barletta et al., 2016; Martins et al., 2018), while others have observed that DENV induces a proviral selective autophagy targeting LDs, named lipophagy (Heaton and Randall, 2011). Recently, a role for AUP1, an LDs associated cell protein, has been well described for this process in DENV infection: viral NS4A and NS4B proteins interact with AUP1 to hijack its acyltransferase function, triggering lipophagy to improve the production of infective particles (Zhang et al., 2018). The authors report that this mechanism appears to be also functional in ZIKV and WNV infections, turning it an apparent general phenomenon for infective flavivirus production. Although an explanation for the discrepancy between the published data has not been found yet, it is possible that these differences are the result of a combination of the cellular system and the high virus-to-cell ratio used to infect monolayers (Samsa et al., 2009; Heaton and Randall, 2010,2011). Moreover, the type of analysis done is not the same in every case, since some authors measure LDs number and total LDs area per cell, while others focus only on LDs number. Given that LDs can vary in size, it is possible that the phenotypes observed correspond to the activation of the same cellular process and that differences arise from the different cell lines and the times of infection used. Also, it cannot be discarded that viruses might induce LDs biogenesis stimulating the initial viral replication, and later on trigger lipophagy decreasing LDs number to release free fatty acids from these lipid structures. Hence, depending on which stage of the viral replication the LDs are measured, different conclusions could be drawn. It is worth to mention that when several viral cycles occur simultaneously on a monolayer, different phenomenon may compete and/or add to the final cell phenotype. Then, we decided to limit our study to 24 h p.i., with few cycles of viral replication, and we found a significantly decreased LDs content in ZIKV infected Huh-7 cells, with reduction in the number of LDs/cell and the LDs volume.

The localization of the capsid C protein of DENV and HCV at LDs organelles has been extensively reported (Shavinskaya et al., 2007; Samsa et al., 2009). Additionally, the localization of the capsid C protein of ZIKV to LDs was documented in HEK 293 (Coyaud et al., 2018), BHK-21 (Shang et al., 2018), and Vero (Hou et al., 2017) infected cells, with LDs representing the main location of C protein in the host cell. Although the mechanism behind the reduced content of LDs in Huh-7 infected cells here reported has not been addressed for ZIKV infection, our results confirm that this virus produces cell phenotypes related to lipid homeostasis comparable to others members of the family.

Collectively, the observations reported here showing a reorganization of three cell components, PML-NBs, mitochondria and LDs, demonstrate the importance of these subcellular structures for proper flavivirus replication, but the *in-vivo* relevance of these results remains unexplored. Several inhibitors targeting host organelles are well characterized. Therefore, a more comprehensive understanding of the molecular biology of viruses and their dependence on host organelles is of utmost priority for development of broad-spectrum and specific anti-flaviviral strategies.

## Data Availability Statement

All datasets generated for this study are included in the article/Supplementary Material.

## Author Contributions

CG, SC, AA, and ED contributed conception and design of the study. CV, FG, and CR performed the experiments and analyzed data. All authors contributed to manuscript revision, read, and approved the submitted version.

## Conflict of Interest

The authors declare that the research was conducted in the absence of any commercial or financial relationships that could be construed as a potential conflict of interest.

## Supplementary Material

FIGURE S1 Single-cell image analysis for LDs quantification. Upper panels: Non-infected Huh-7 monolayer. Lower panels: ZIKV- infected Huh-7 monolayer. Augmentation: 60×. **(A,I)** Confocal plane of the green channel (ZIKV-E protein) in non-infected and infected cultures. Inset: Histogram of each image, showing that even though there is no visible image in non-infected cultures, autofluorescence signal from cells can be detected. **(B,J)** After being processed with a Gaussian Blur filter, the pattern present in the microphotographs is smoothed, simplifying whole-cell thresholding. **(C,K)** Automatically selected areas in each photograph by using the mean of gray levels of the stack as the threshold. **(D,L)** Final binary masks obtained after refining the thresholding output, by using the “Fill holes” and “Watershed” algorithms. **(E,M)** ROIs corresponding to individual cells, as obtained after using “Analyze Particles” plugin. **(F,N)** Confocal plane of LipidTox-stained LDs in both, infected and non-infected cultures. **(G,O)** LDs area obtained with “3D Object Counter” plugin. **(H,P)** LDs selected with “Find Maxima” plugin for LDs number quantification.
