# Supplementary figures and images for: Cellular Organelles Reorganization During Zika Virus Infection of Human Cells
Source: Front Microbiol. 2020 Jul 8;11:1558. doi: 10.3389/fmicb.2020.01558 (PMC7381349; doi:10.3389/fmicb.2020.01558)

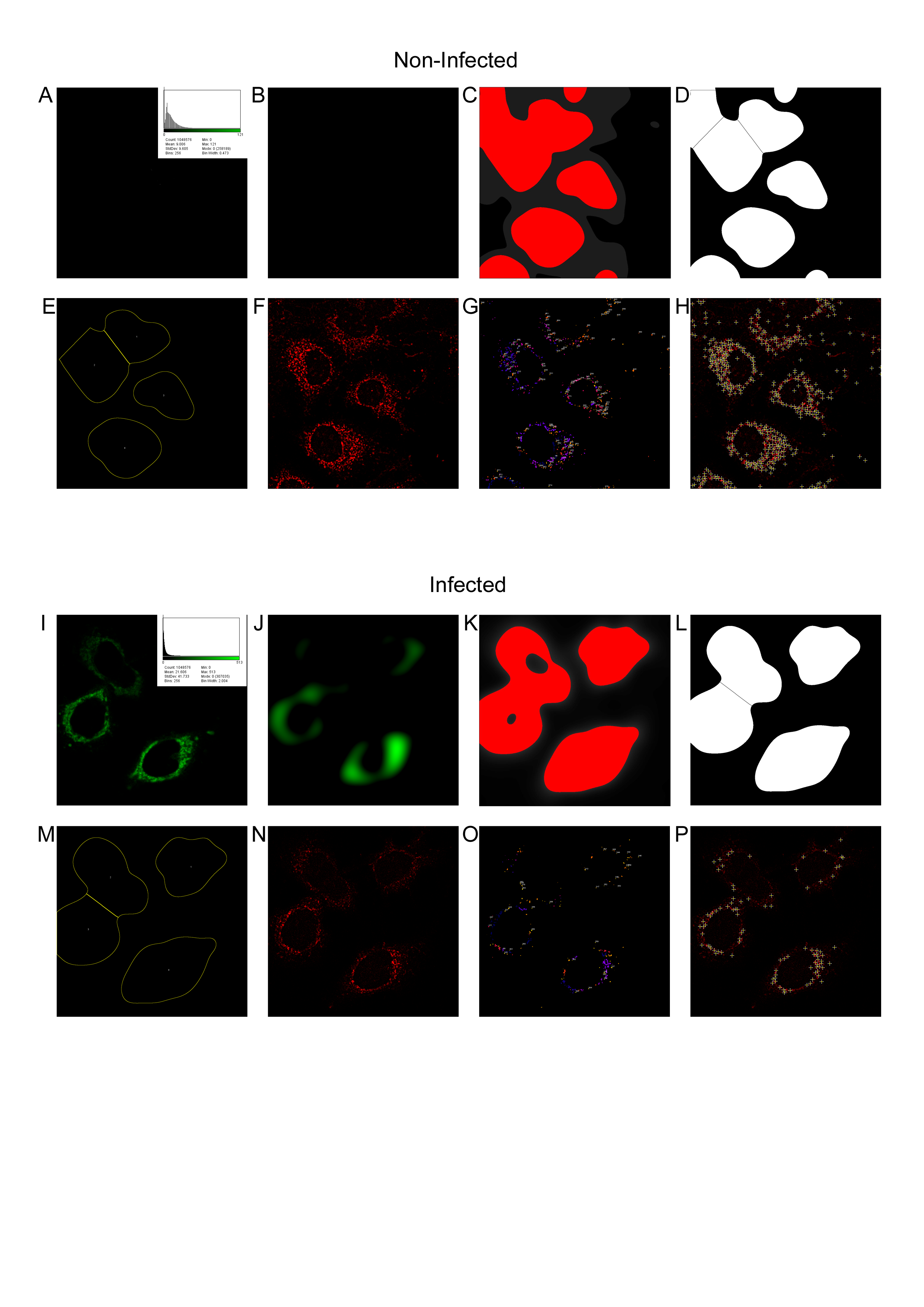

Supplement: FIGURE S1 — Single-cell image analysis for LDs quantification. Upper panels: Non-infected Huh-7 monolayer. Lower panels: ZIKV- infected Huh-7 monolayer. Augmentation: 60×. (A,I) Confocal plane of the green channel (ZIKV-E protein) in non-infected and infected cultures. Inset: Histogram of each image, showing that even though there is no visible image in non-infected cultures, autofluorescence signal from cells can be detected. (B,J) After being processed with a Gaussian Blur filter, the pattern present in the microphotographs is smoothed, simplifying whole-cell thresholding. (C,K) Automatically selected areas in each photograph by using the mean of gray levels of the stack as the threshold. (D,L) Final binary masks obtained after refining the thresholding output, by using the “Fill holes” and “Watershed” algorithms. (E,M) ROIs corresponding to individual cells, as obtained after using “Analyze Particles” plugin. (F,N) Confocal plane of LipidTox-stained LDs in both, infected and non-infected cultures. (G,O) LDs area obtained with “3D Object Counter” plugin. (H,P) LDs selected with “Find Maxima” plugin for LDs number quantification. [file Image_1.TIF]
